# Assessing clogging of laminated hydrophobic membrane during fecal sludge drying

**DOI:** 10.1016/j.scitotenv.2018.01.209

**Published:** 2018-06-15

**Authors:** Babak Ebrazi Bakhshayesh, Paul T. Imhoff, Steven K. Dentel

**Affiliations:** Department of Civil and Environmental Engineering, University of Delaware, 344A DuPont Hall, Newark, DE 19716, USA

**Keywords:** Fecal sludge drying, Laminated hydrophobic membrane, Clogging, Container-based sanitation systems

## Abstract

A new sanitation technology has been proposed in which a laminated hydrophobic membrane contains and enhances drying of fecal sludge in a toilet, with particular focus on application to urban regions of low-income countries. The proposed technology uses a laminated hydrophobic membrane liner as an integral component of container-based sanitation systems. The focus of this study is to quantitatively evaluate the laminate's clogging after repeated use, which will affect replacement interval and might limit the laminate's application in container-based toilets. The membrane of the laminated hydrophobic membrane used in this process is hydrophobic and only allows vapor transport. Drying of water vapor using the laminated hydrophobic membrane occurs due to moderate temperature or humidity gradients, while other constituents such as aqueous dissolved solutes of fecal sludge are retained. Controlled laboratory experiments evaluated repeated use of a laminated hydrophobic membrane for fecal sludge drying, with mild brushing/rinsing of the laminate between each application. Drying occurred at a constant rate as long as the fecal sludge moisture content exceeded 11.6 (g/g), below which water activity <1. Over five drying cycles, at a significance level of *α* = 0.05 the dimensionless drying rate in the constant-rate period was not reduced. While scanning electron microscopy and energy dispersive X-ray analyses of used laminated hydrophobic membrane showed deposition of fecal sludge on the inner fabric of the laminate, particulate accumulation was never sufficient to alter the fecal sludge drying rate. Experiments with only water indicated that the fecal sludge increased the effective diffusion length through the laminate by 10–30%. These data demonstrate that clogging of the laminated hydrophobic membrane is minor over five cycles of fecal sludge drying with mild rinsing between cycles, indicating that use of the laminate may be feasible in many field applications.

## Highlights

• A laminated hydrophobic membrane permitted significant fecal sludge (FS) drying over 5 cycles.
• Particulate accumulation from FS between cycles occurred but did not reduce drying.
• FS drying rate decreased below a critical moisture content, when water activity <1.
• FS drying was 10–30% slower than water evaporation through laminated hydrophobic membrane.

## Graphical abstract

**Figure:**
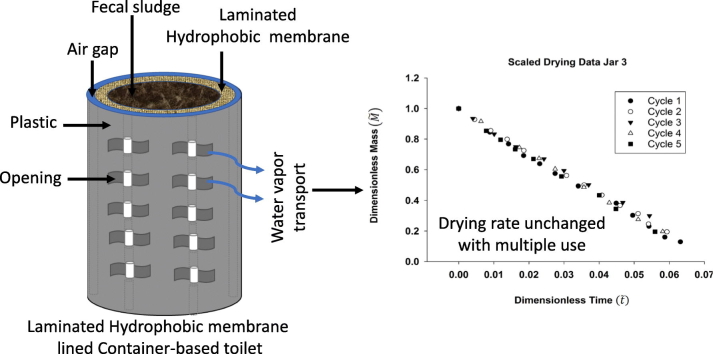

## 1 Introduction

According to the World Health Organization and UNICEF only 63% of the world's population has access to improved sanitation facilities that eliminate human contact with fecal sludge (FS) (WHO, 2013). FS is a combination of human feces, urine and blackwater, and may contain greywater (Strande et al., 2014). An estimated 2.8 billion people live without adequate sanitation, 85% of whom use unimproved sanitation facilities where hygienic separation of FS from human contact is not ensured. Unimproved sanitation systems include unlined pit latrines, while approximately 15% of those living without adequate sanitation practice open-defecation (Katukiza et al., 2012; Mara et al., 2010; UNDP, 2006). Inadequate sanitation systems in urban slums are a factor causing diarrhea and bacterial infections, which result in 1.5 million child deaths per year (Mara et al., 2010; Prüss-Üstün et al., 2008; Sidhu and Toze, 2009; WHO, 2013). The lack of appropriate sanitation technologies is one reason why open defection is widely practiced in low-income countries (WHO, 2013).

In urban slums, pit latrines are a widely-used sanitation technology (Chaggu et al., 2002; Howard et al., 2003; Kulabago et al., 2009; Thye et al., 2011; WHO, 2013). Pit latrines consist of a hole in the ground where FS is collected, with fluids infiltrating from the hole into the subsurface. In 2013, an estimated 1.8 billion people were reported to utilize pit latrines as the primary means of FS collection (Graham and Polizzotto, 2013). Even if pit latrines are properly constructed, operated, maintained and emptied to prevent solids buildup, they often result in environmental and health risks in urban slums (Howard et al., 2003; Kulabago et al., 2009). Firstly, pit latrines are usually emptied manually due to the narrow streets in dense urban slums that make them inaccessible to waste removal trucks. This emptying practice may expose laborers and community residents to FS pathogens and parasites (Russel et al., 2015). Secondly, the aqueous phase of FS collected in pit latrines contains pathogens, parasites and organic contaminants that will infiltrate into the ground and may contaminate groundwater. Finally, pit latrines may overflow during the rainy season with devastating effects on surface water quality (UNDP, 2006). Studies evaluating the health and environmental risks of pit latrines have identified human exposure to microbial and chemical contamination as the main concern for their application (Banerjee, 2011; Dzwairo et al., 2006; Graham and Polizzotto, 2013; Pujari et al., 2012; Verheyen et al., 2009; Vinger et al., 2012; Zingoni et al., 2005).

Although alternatives to pit latrines have been proposed in the developing world in the last decade, finding alternatives in urban slums is challenging due to the lack of money, space, and access, as well as a sense of ownership that is often absent in public facilities (Tobias et al., 2017). Biogas latrines and the Urine Diverting Dry Toilet (UDDT) have been suggested as replacements to pit latrines in urban areas (Katukiza et al., 2010; Katukiza et al., 2012). Biogas latrines utilize anaerobic digestion to produce biogas from FS, which is stored under a fixed dome. The application of biogas latrines has faced some difficulties, though, which include availability of trained personnel for process control, the occurrence of pathogens in the digestate, and high investment costs (Katukiza et al., 2012). UDDTs have faced acceptability issues in some urban slums (Katukiza et al., 2010).

As an alternative to biogas latrines and standard UDDTs, a container-based sanitation (CBS) system was recently proposed for use in urban slum areas where constructing sewerage is infeasible (Tilmans et al., 2015). In a CBS system, FS is collected in a sealable container that is then transported to a treatment facility, emptied, and returned and reused for FS collection. Many CBS systems may employ urine-diverting toilets, and thus might be considered a type of UDDT (Tilmans et al., 2015). Infrastructure associated with FS collection in a CBS system is typically situated above ground, which reduces construction costs and the system's vulnerability to flooding. In a pilot-scale study in Haiti where the feasibility of CBS systems were evaluated, the main advantage of the CBS system was less human exposure to FS, malodor and insects, since FS was stored and transported in sealed containers (Tilmans et al., 2015). The CBS system had the advantage of 24-h accessibility (Russel et al., 2015), and users were sufficiently satisfied such that 71% planned to become paying subscribers after the completion of the free testing period (Russel et al., 2015). However, the pilot-scale study also found that the operational costs of the CBS system was approximately two to three times more than operational costs for large-scale conventional waterborne sewerage collection (Russel et al., 2015). Most of this additional cost was associated with labor for emptying containers. Since water is the major component of FS, a technology that lowers the moisture content of FS during CBS system usage will decrease the system's operational costs by reducing the frequency of emptying. Alternatively, such a technology would lower the weight of CBS containers and thus lower transportation costs, and likely costs associated with final disposal of FS.

To reduce labor costs of CBS systems, a low-cost and sustainable solution was recently proposed (Marzooghi et al., 2017). In this approach, the membrane distillation process was adapted to biosolids drying in CBS system toilets by enclosing biosolids in a laminated hydrophobic membrane; the laminated hydrophobic membrane allows biosolids drying while preventing transport of aqueous dissolved solutes from biosolids. This characteristic depends on the hydrophobicity and pore structure of the laminated hydrophobic membranes (Marzooghi et al., 2017). The mass transfer of water vapor across a laminate is analogous to membrane distillation in which a vapor pressure gradient is the driving force for water vapor transport. While in membrane distillation the temperature difference between the feed and the permeate sides of the membrane controls the vapor pressure gradient (Curcio and Drioli, 2005; Souhaimi and Matsuura, 2011), when a laminated hydrophobic membrane encloses biosolids the drying rate is also controlled by the relative humidity (*RH*) of air external to the laminate. When the *RH* of external air is high and near 100%, the drying rate is reduced, but when *RH* is small it is enhanced.

To evaluate if lining toilets in CBS systems with laminated hydrophobic membranes might be efficacious, anaerobically digested sludge, i.e. biosolids, were enclosed in a commercially available laminated hydrophobic membrane, the GORE Wrap Cover Laminate (W.L. Gore & Associates, Inc., DE, USA). In a series of drying experiments, the biosolids' moisture content decreased from 97% to 12–30% while the transport of fecal coliforms across the laminated hydrophobic membrane was negligible. Moreover, the concentration of fecal coliforms in biosolids decreased three orders of magnitude during biosolids drying, which indicated fecal coliforms were inactivated during the drying process. At the end of the drying experiment, the concentration of fecal coliforms in biosolids met Class A and Class B requirements (USEPA, 2002).

While Marzooghi et al. (2017)’s results indicate that laminated hydrophobic membranes enhance biosolids drying and thus could reduce the frequency of toilet emptying in CBS systems, there are three unanswered questions regarding the application of this technology in a CBS sanitation system. First, Marzooghi et al. (2017) conducted their experiments using anaerobically digested biosolids, but will waste drying be different for FS since the chemical, physical and biological characteristics of FS differ for biosolids? For example, organic matter, total solids, ammonium, and helminth egg concentrations in FS are typically a factor of ten or a hundred larger than in biosolids from wastewater sludge (Strande et al., 2014). The dissimilar composition of the aqueous phase in FS and biosolids may result in different water activities in these wastes and water vapor transfer rates across laminates during drying.

Second, will water vapor permeation through the laminated hydrophobic membrane change significantly with repeated use of a laminate given that the Marzooghi et al. (2017) study consisted of a single cycle of biosolids drying? FS contains large colloidal organic and inorganic compounds (Strande et al., 2014) that may accumulate on the fabric of a laminate, resulting in additional resistance to water vapor transport and lower water vapor transfer rate with repeated laminate use. If significant, such clogging would deteriorate performance of a CBS system and increase cost of operation, if frequent cleaning or laminate replacement is needed.

Finally, while Marzooghi et al. (2017) evaluated the GORE Wrap Cover laminate for biosolids' drying, might other laminated hydrophobic membranes perform better, particularly those with hydrophilic protective fabrics? There are many commercially-available laminated hydrophobic membranes, including the eVent™ (eVent® fabrics, Overland Park, USA), Entrant GII XT (Toray Industries, New York, NY, USA), Gore-Tex XCR and Gore-Tex (Gore-Tex®, Newark, DE, USA), Dermizax (Toray Industries, New York, NY, USA), Sympatex (Akzo-Nobel, Amsterdam, Netherlands), and Conduit (Mountain Hardware, Richmond, CA, USA) laminates. While these products are similar and consist of an expanded porous hydrophobic membrane sandwiched between two fabrics, they have different water vapor transfer rates. A study examining the vapor transport properties of protective clothing layers evaluated water vapor transfer in 15 products including several laminated hydrophobic membranes (Gibson and Schreuder-Gibson, 2009). Among the laminates tested, the eVent laminate had the greatest water vapor transfer rate, with water vapor flux approximately 5100 g/m^2^/day, which was approximately 19% higher than that of the second best laminate (Gibson and Schreuder-Gibson, 2009). While both protective fabrics covering the hydrophobic membrane in the GORE Wrap Cover laminate tested by Marzooghi et al. (2017) are hydrophobic, one of these fabrics for the eVent laminate is hydrophilic and if in contact with FS may result in vapor phase diffusion paths that are significantly shorter than in the GORE Wrap Cover laminate.

The primary objective of this study was to address these three questions for drying of FS retained in a laminated hydrophobic membrane: Can the same relatively rapid drying achieved for biosolids also be achieved for FS? Does FS drying diminish significantly with reuse of the laminate, if only mild cleaning is employed between applications? Is the eVent laminate, which was identified as possessing the highest water vapor transmission rate in a recent study and includes a hydrophilic protective fabric, significantly better than the previously tested GORE Wrap Cover laminate (Marzooghi et al., 2017)? Finally, experiments were also conducted to evaluate if drying through the eVent laminate is significantly affected by height of FS, which will vary depending on the design and mass of stored FS in a laminate-lined toilet. Although, laminates possess high tensile and tear strength that easily sustain the weight of FS, as FS mass increases higher water pressures against the laminate may alter FS drying.

### 1.1 Overview of drying process of FS

**Fig. 1:**
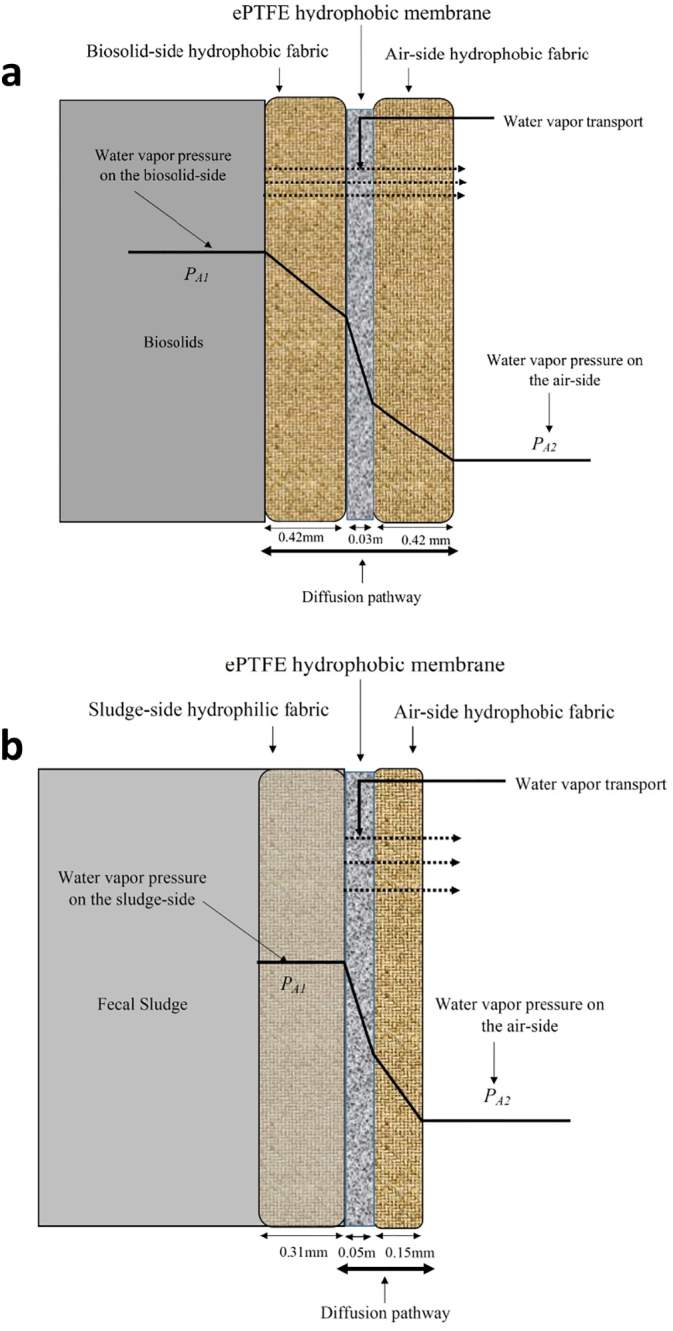
Schematic of water vapor pressure gradient between biosolids and air-side of GORE Wrap Cover laminate (a), and between FS and air-side of eVent laminate (b). *P*_*A1*_ and *P*_*A2*_ are the water vapor pressures on the biosolids/FS side and the air-side, respectively.

The GORE Wrap Cover laminate tested by Marzooghi et al. (2017) consists of three separate pieces (Fig. 1a): an inner microporous expanded polytetrafluorethylene (ePTFE) porous hydrophobic membrane, and two outer woven hydrophobic fabrics sandwiching and protecting the membrane. The eVent laminate is similar, except one of the outer woven fabrics is hydrophobic and the other hydrophilic (Fig. 1b). When the laminates enclose FS, pores in the hydrophobic fabric of the GORE Wrap Cover laminate remain air-filled, while pores in the hydrophilic fabric of the eVent laminate that contact FS may be water-filled.

**Fig. 2:**
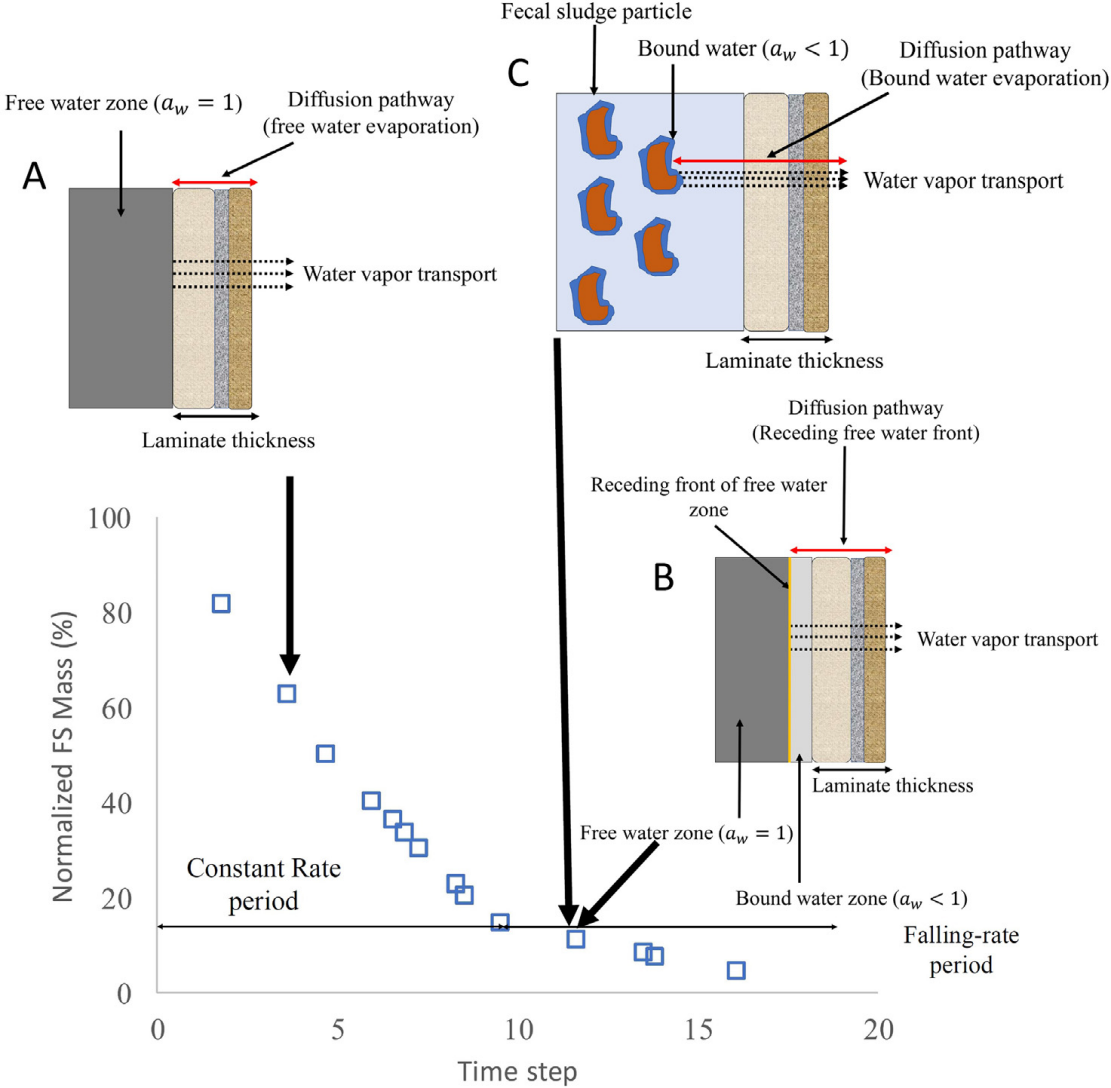
Two periods of FS drying: 1) constant-rate drying period (A: free water evaporation), and 2) falling-rate drying period (B: free water front is away from the laminate, and/or C: primarily bound-water evaporation).

The drying of FS retained in laminated hydrophobic membranes occurs in two distinct periods as shown in Fig. 2 – a constant-rate and a falling-rate drying period (Marzooghi et al., 2017). During the constant-rate drying period, evaporation of free water within FS occurs (Fig. 2A). Constant-rate drying is controlled by the temperature and *RH* of the environment to which FS is exposed. Throughout the constant-rate period, the water vapor pressure within FS is assumed equal to the saturation vapor pressure, due to the presence of free water within FS, and water activity ( *a*_*w*_) = 1 (Vaxelaire and Cézac, 2004). The second period is characterized by a decreasing drying rate due to two factors: (1) free water recession within FS farther away from the laminate, which increases water vapor diffusion length (Fig. 2B); or (2) free water no longer present in FS, such that primarily bound water evaporates during drying (Fig. 2C) (Vaxelaire and Cézac, 2004). It is postulated that once the moisture content of FS drops below a critical value, *a*_*w*_ at the laminate/FS interface becomes less than one. Under these conditions, *a*_*w*_ and consequently water vapor pressure continues to decrease with decreasing FS moisture content and water vapor pressure remains below the saturated water vapor pressure, reducing the driving force for diffusion across the laminate (Vaxelaire and Cézac, 2004). If mechanism (2) controls the decreasing drying rate of FS, the critical moisture content marks the transition from the constant-rate to falling-rate drying periods (Vaxelaire and Cézac, 2004).

During laminated hydrophobic membrane drying of FS in CBS systems, it is postulated that the constant-rate period best describes FS drying under most conditions, and that diffusive transport of water vapor through the laminate controls the rate of moisture loss. A schematic of water vapor diffusion through the laminated hydrophobic membrane for this situation is shown in Fig. 1. Because of the hydrophobic membrane, laminated hydrophobic membranes block the passage of biosolids' aqueous phase while allowing water vapor transfer. Factors controlling water vapor diffusion through a laminate include gradients of *RH* and temperature, and the laminate's resistance to diffusive transport of water vapor. The total resistance for diffusive water vapor transport through the laminate includes resistance to transport by each layer of the laminate.

For transport through a hydrophobic membrane where pore sizes are submicron, both molecular diffusion and Knudsen diffusion may be important (Alkhudhiri et al., 2012). However, Marzooghi et al. (2017) showed that for the Gore Wrap Cover laminate transport was dominated by molecular diffusion through the inner and outer fabric, such that Knudsen diffusion through the inner, hydrophobic membrane could be neglected. A similar analysis as described by Marzooghi et al. (2017) was conducted for the eVent laminate used in this work: neglecting Knudsen diffusion results in less than a 2% error in predicted moisture transfer through the eVent laminate. For this situation, molecular diffusion of water vapor across a three-component laminate controls vapor transport and can be described by steady-state diffusion of water through a stationary film of air trapped within laminate pores as depicted in Fig. 1a (biosolids, Gore Wrap Cover laminate) and Fig. 1b (FS, eVent laminate):

(1) $$J_{A}=\frac{PD_{\mathrm{AB}}}{\mathrm{R\lambda}\left(T_{\mathrm{avg}}+273.15\right)}\;\ln\left(\frac{P-P_{A1}}{P-P_{A2}}\right)$$

where *J*_*A*_ (moles/m^2^ s) is the flux of component *A* (water vapor) through the stagnant air film, *P* (Pa) is the average total gas pressure in the film and is often equal to atmospheric pressure evaluated at *T*_*avg*_, and *R* (m^3^ Pa mol^−1^ K^−1^) is the ideal gas constant. *T*_*avg*_ (°C) is the average of *T*_*1*_ (°C) and *T*_*2*_ (°C), temperatures on the sludge side and air side of the laminate, respectively. *D*_*AB*_ (m^2^/s) is the diffusivity of component *A* in component *B* (air) evaluated at *T*_*avg*_, $\lambda =\frac{\tau \delta }{\varepsilon }$ (m) is an effective diffusive length that accounts for the thickness (*δ*), dimensionless tortuosity (*τ*), and dimensionless porosity (*ε*) of the three-component laminate, and *P*_*A1*_ and *P*_*A2*_ (Pa) are the water vapor pressures on either side of the laminate. *D*_*AB*_ is a function of temperature and for the diffusion of water vapor in air can be estimated with (Gibson, 2000):

(2) $$D_{\mathrm{AB}}=\left(2.23\times 10^{-5}\right){\left[\frac{T_{\mathrm{avg}}+273.15}{273.15}\right]}^{1.75}$$

Water vapor pressure on both sides of the laminate is estimated using *RH* and temperature of each side. The vapor pressure at the saturated state is appropriate for the FS side of the laminate during the constant-rate drying period and can be estimated with Antoine's equation (Poling et al., 2001) where *T* (°C) is the FS temperature:

(3) $$\;p_{A,\mathrm{sat}}=\exp\left[23.238-3841/\left(T+228.51\right)\right]$$

### 1.2 Scaling drying data

FS drying with a laminated hydrophobic membrane occurs naturally because of water vapor diffusion without any application of external heat, if ambient *RH* is <100%. Since ambient conditions vary with time and may be difficult to control during and between experiments, a dimensionless form of Eq. (1) was developed to describe FS drying for experiments reported in this study:

(4) $$\overset{\sim }{M}=\frac{\varepsilon }{\tau }\;\overset{\sim }{t}+1$$

where $\overset{\sim }{M}$ and $\overset{\sim }{t}$ are the dimensionless FS mass inside the laminate enclosure and dimensionless time, respectively, and are defined:

(5) $$\overset{\sim }{M}=\frac{M}{M_{i}}$$

(6) $$\overset{\sim }{t}=\frac{M_{w}\;\mathrm{SA}\;P\;D_{\mathrm{AB}}}{M_{i}\;R\;\left(T_{\mathrm{avg}}+273.15\right)\delta }\ln\left(\frac{P-P_{A1}}{P-P_{A2}}\right)t$$

where *M* (g) is the mass of FS in the laminate enclosure, *M*_*i*_ (g) is the mass at the start of the experiment, *M*_*w*_ (g/mol) is the molecular weight of water, *SA* (m^***2***^) is the outer surface area of the laminate, and *δ* (m) is the thickness of the laminate. Experimental data reported below were scaled using Eqs. (5), (6) and then regressed according to Eq*.* (4) to determine *ε*/*τ*. This slope is independent of internal and external environmental conditions and was used to determine any changes in laminate water vapor transfer capability due to clogging.

## 2 Material and methods

### 2.1 Collection of fecal sludge

Three FS samples were used in this study. Batches #1 and #2 were collected from three males who had a Mediterranean diet that consisted primarily of fruits, vegetables, grains, and olive oil, which is the main source of fat, dairy products, and fish that is consumed in low to moderate amounts (Willett et al., 1995). Batch #3 was collected from undergraduate student volunteers at the University of Delaware, who did not have a specific diet. FS samples were collected using a flushable portable toilet. The toilet includes a small plumbing system with a waste-holding tank that collects feces, urine, and wash water without separation.

Batch #1 was collected over one week and stored at 3–4 °C in a sealed plastic container refrigerated for approximately two months prior to experiments. Batch #2 was collected from the same donors during a different one-week period and stored in a similar fashion for approximately seven days before experiments. Batch #3 was collected and stored in a similar fashion for less than three months before experiments. Batch #1 was used in FS drying and water activity measurements, while Batch #2 was only used for water activity measurements. Batch #3 was utilized in scanning electron microscopy experiments.

### 2.2 Fecal sludge water activity

A water desorption isotherm describes, at a fixed temperature, the evolution of the thermodynamic activity of the water versus moisture content within FS. The desorption isotherm was determined by the chilled mirror dewpoint method, which is based on equilibrating the aqueous phase water in the sample with vapor phase in the headspace of a closed chamber and measuring *RH* of the headspace (Vaxelaire, 2001). From thermodynamics and under some assumptions, *a*_*w*_ is equal to measured *RH* under equilibrium conditions (Vaxelaire and Cézac, 2004).

To obtain the desorption isotherm for FS, one well-mixed 250 mL FS sample from Batch #1 and three well-mixed replicate 250 mL FS samples from Batch #2 were used. The initial moisture contents of these slurries were 27.4 ± 1.8 (95% confidence interval) (g/g) and 28.3 ± 2.1(g/g), for Batch #1 and #2, respectively. Samples were dried slowly in an oven at a temperature of 105 °C to different moisture contents. At selected times during oven-drying, well-mixed subsamples with an approximate weight of 5 g were collected. One well-mixed subsample was taken from Batch #1, and a well-mixed subsample was collected from each of the three Batch #2 replicates and stored in sealed plastic containers. The water activity of each subsample was then measured with an AquaLab CX-2 water activity meter (AquaLab, Pullman, WA, USA), followed by oven drying at 105 °C for 24 h to obtain the corresponding moisture content of the subsamples. These data were used to determine the relationship between water activity and moisture content for FS.

### 2.3 Fecal sludge drying

All FS drying experiments were conducted using the eVent laminate (Fig. 1b) (eVent® fabrics, Overland Park, USA). This laminate contains a gas permeable, hydrophobic ePTFE membrane and is supported by a 0.15 mm thick hydrophobic 300D polyester fabric on one side and a hydrophilic 20D mesh-like tricot layer backing fabric on the other side. The average pore size of the membrane is 0.14 μm as determined by BET analysis (Micromeritics ASAP 2020 analyzer), while the membrane thickness was estimated from scanning electron microscopy (Hitachi S-4700) and is 46.4 μm.

The eVent laminate is analogous to the Gore Wrap Cover laminate tested by Marzooghi et al. (2017): both contain a microporous hydrophobic ePTFE membrane sandwiched between two fabrics (Marzooghi et al., 2017). The average pore size of the ePTFE membrane in the Gore laminate is 0.2 μm (Marzooghi et al., 2017), which is larger than the 0.14 μm average pore size of the eVent laminate's membrane. However, the eVent laminate is thinner, 0.5 mm versus 0.87 mm, and has an inner hydrophilic and outer hydrophobic fabric, while both fabrics protecting the ePTFE membrane in the Gore Wrap Cover laminate are hydrophobic (see Fig. 1). Since vapor diffusion through the laminate is dominated by resistance through the fabrics (Marzooghi et al., 2017), if the hydrophilic side of the eVent laminate is in contact with wet FS, drying is expected to be faster for the eVent laminate than the Gore Wrap Cover. Experiments reported below tested this hypothesis.

Drying experiments were performed in a Conviron model No. PGC 20 growth chamber (Pembina ND, USA). The growth chamber was used to maintain the ambient temperature and *RH* at 31.4 ± 0.8 °C and 30.9 ± 1.4%, respectively.

**Fig. 3:**
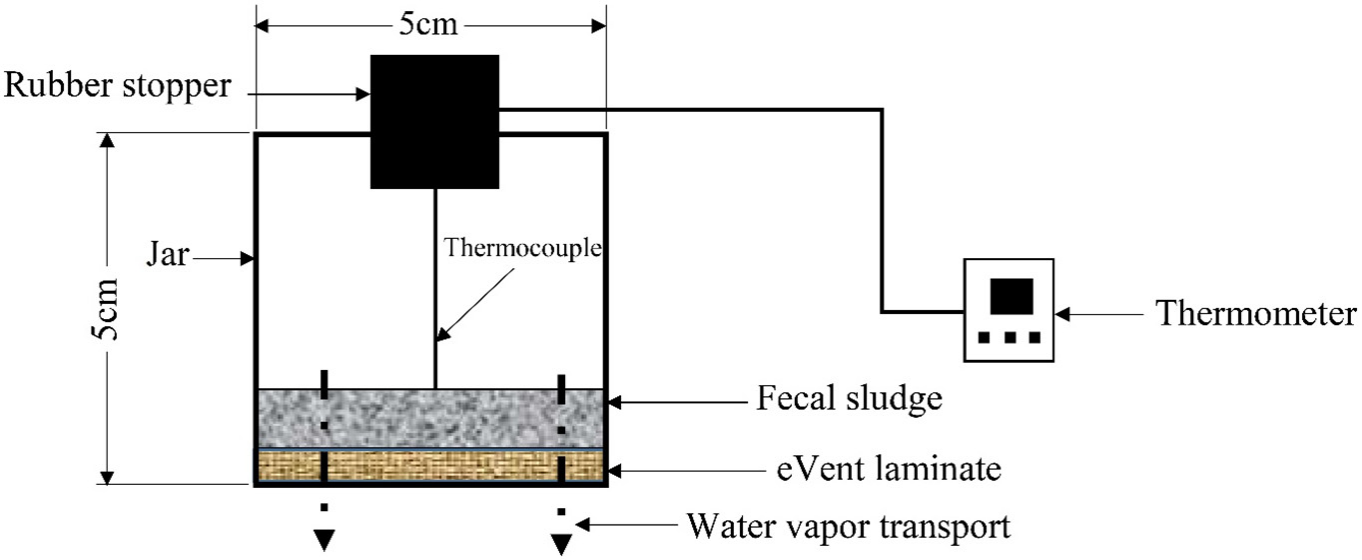
Schematic of experimental setup used for FS drying, which is a laminated hydrophobic membrane-covered cylindrical glass jar. The bottom of the jar was drilled and sealed with a rubber stopper, while its metal lid was replaced with eVent laminate before inverting the jar. A thermocouple was used to record the temperature of FS contained in the jar.

To determine any reduction in performance of the eVent laminate due to repeated use for FS drying, the experimental setup shown in Fig. 3 was used. The setup contained a glass canning jar with a diameter and height of 5 cm whose top was replaced with the eVent laminate sealed onto the glass jar with silicon glue (GE Silicone II, Momentive Performance Materials Inc., Huntersville, NC, USA). The surface area of eVent laminate through which water vapor diffusion occurred was 20 cm^2^. A 2-cm diameter hole was cut in the bottom of the jar that was used for filling, emptying, and cleaning the jar. This opening was sealed tightly with a rubber stopper when not in use. Prior to the drying experiments, the jars were filled with water, inverted such that the rubber stopper was in contact with a paper towel, and observed for 1 h to confirm no water leakage. To ensure FS was in direct contact with the laminate during drying experiments, once filled with FS jars were positioned upside down on top of a metal frame that permitted air flow around the laminate during drying.

FS drying experiments were conducted over five cycles. For each cycle four identical jars were utilized: three filled with 15 mL of Batch #1, while the fourth was filled with the same volume of deionized water (DI). This volume formed a 4–5 mm layer of FS or DI water on top of the laminate. After the initiation of drying in each cycle, jars were weighed periodically to 0.01 g (Mettler Toledo scale XP4002S, Switzerland), and their inside temperature was recorded without opening the jar with a thermometer (Omega RDXL4SD, Stamford Connecticut, USA) with ±0.5 °C precision whose thermocouple was permanently in contact with FS or DI water inside each jar. This thermocouple was inserted through a ~2 mm diameter hole through the stopper and sealed with vacuum grease. Temperature measurements were made approximately 0.5 mm from the laminate surface. *RH* and temperature outside of the jars but inside the growth chamber were recorded every 1 min with a *RH*/temperature data logger (EL-USB-2-LCD, MicroDAQ.com, Ltd., Erie, PA, USA) with ±3.0% and 0.5 °C precision for *RH* and temperature, respectively. The drying process for each cycle was stopped when the mass change of the jars between successive sampling times was <0.01 g.

At the end of each cycle, rubber stoppers were removed from each jar, dried FS deposited on the laminate was gently brushed off, the laminate was rinsed with DI water, and finally the laminate was gently brushed again. This cleaning process was meant to mimic mild cleaning that may occur during CBS system operations, where laminates may be installed as integral components of containers collecting FS. Finally, jars were refilled with the same volume of FS (Batch #1) or DI water for the next drying cycle.

### 2.4 Scanning electron microscopy and energy dispersive X-ray analyses

The eVent laminate was analyzed to assess particle buildup after a five cycle FS drying experiment. The setup used in this experiment was constructed by gluing the eVent laminate to a rigid plastic, envelope-like enclosure with top half covered with flexible laminate and a bottom plastic half. This setup is nearly identical to that described in more detail in Marzooghi et al. (2017). Four such enclosures were used, one of which was filed with 100 mL DI water, while the other three were filled with 100 mL of FS from Batch #3 with an initial FS depth of about 6 cm inside the enclosure. Drying experiments were conducted in a constant temperature room at 30 °C and repeated for five cycles. At the end of each cycle, FS residues were removed gently from the surface of the laminate for one of the enclosures by gentle brushing followed by DI water rinse, and then refilled with 100 mL FS (used/rinsed). However, the other two FS-filled enclosures were not brushed and rinsed (used/unrinsed). At the end of cycle 5, the used/rinsed and used/unrinsed laminates were removed and sampled at two locations: middle and edge. The area of each laminate sample was approximately 1 cm^2^. Similar samples were collected from the laminate filled with DI water(new) for comparison.

Scanning electron microscopy (SEM) and energy dispersive X-ray (EDX) analyses were performed on these laminate samples using a Hitachi S4700 FE-SEM at 20 Kv with an EDX analyzer (SEM Hitachi S-4800, Japan). Samples were attached to SEM aluminum stubs and coated with Au/Pd or Carbon in a Denton Vacuum Turbo Top III sputter-coater.

### 2.5 Water pressure effect on drying rate

The experimental setup shown in Fig. 3 was modified to evaluate the effect of water pressure on drying rate through the eVent laminate. To create a prescribed water pressure on the laminate, a 1 cm diameter hole was made in the rubber stopper through which a glass tube was inserted. The jar was filled with DI water and added to this tube at a prescribed height above the laminate that varied with experiment: 10, 30, 40, 60, 90 and 120 cm of water. To determine the effect of water pressure on drying rate, drying rates at the prescribed water pressure heads (10, 30, 40, 60, 90 and 120 cm) were compared to that of 1 cm water, which was created by covering the laminate with 1 cm of DI water and sealing the jar with a rubber stopper. In order to conduct experiments at the same ambient conditions (temperature and *RH*), all drying experiments were conducted simultaneously in different jars for 4 h. Changes in water height in the glass tube over each 4 h test were used to determine water mass losses and drying rates at the 10, 30, 40, 60, 90 and 120 cm pressure heads, while the drying rate for 1 cm water height was determined by weighing the jar before and after the 4 h test (Mettler Toledo scale XP4002S, Switzerland).

## 3 Results and discussion

### 3.1 Fecal sludge water activity

**Fig. 4:**
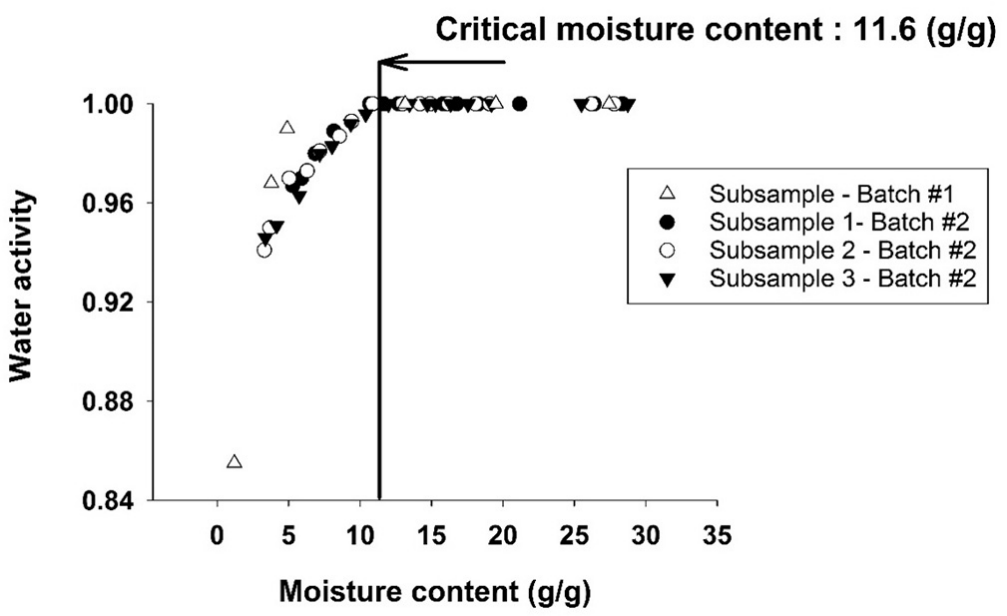
Water activity of FS at different moisture contents determined during desorption experiments.

Water activity measurements of FS are shown in Fig. 4. The initial moisture content of FS ranged from 25 to 30 (g of water/g of dried solid) and decreased to 2–5 (g/g) over the course of oven drying. The water activity was 1.0 for all FS samples until the moisture content decreased below 11.6 ± 0.72 (g/g). Because *a*_*w*_ = 1 corresponds to conditions of free water within FS (Vaxelaire, 2001), over a wide moisture content range (30 to 11.6 (g/g)) free water existed and a constant FS drying rate is anticipated. Even below the critical moisture of 11.6 (g/g), FS water activity remains above ~0.94 until the moisture content decreases below ~4 (g/g). Thus, for FS enclosed by a laminate, saturated water vapor conditions are expected to exist at the inner laminate fabric (see Fig. 1) for extended periods of FS drying.

### 3.2 Fecal sludge drying

**Fig. 5:**
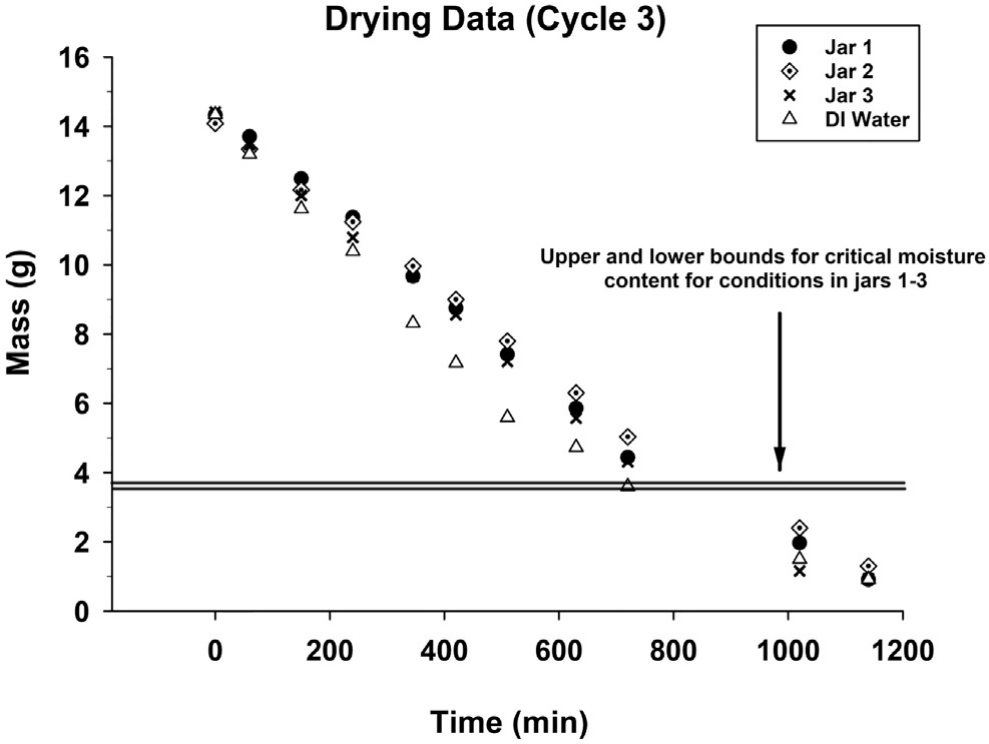
Typical FS drying curves for three replicate jars filled with FS and a reference jar with DI water. Results are shown for drying Cycle 3 conducted at *RH* = 30% and *T* = 30 °C. Horizontal lines bound conditions where moisture contents of FS dropped below the critical value (11.6 g/g).

Typical drying curves are shown in Fig. 5 for the third cycle (Cycle 3) of the drying experiments, where three jars containing wet FS and one jar filled with DI water dried over a 20 h period. FS dried at a linear rate until data dropped below the critical FS moisture content. Below this critical value, water activity and *RH* of FS dropped below 1.0 and drying rates decreased. The drying rate of the control jar, which contained DI water, is faster than that for FS samples, even when moisture content is above the critical value and *a*_*w*_ = 1 for FS. This could be due to clogging of laminate pores with solids from previous drying cycles, nonuniform moisture content within FS such that *a*_*w*_ < 1 at the laminate/FS interface, or other factors.

When sample masses dropped below ~5 g, drying rates for both DI and FS samples decreased. For a 5 g sample of DI water, the calculated water depth over the laminate is ~0.2 mm. Since the laminate is not firm, it deflects and ponding water at shallow depths may not completely cover the laminate surface. This seems the most plausible cause for the decreasing drying rate for DI water and may have been a factor causing the decreasing drying rates for FS samples too.

Drying curves for the other four cycles are similar to those shown in Fig. 5, and data are summarized in Table 1. From the start of drying to the critical moisture content where *a*_*w*_ < 1, the average water mass loss ranged from 68% (Cycle 3) to 77% (Cycle 5) for the three FS samples (Jar 1, 2 and 3) in each cycle. Thus, a significant fraction of the moisture within FS was removed during the constant drying rate period.

**Table 1:** Average water mass loss, moisture content and water activity of FS at the end of the constant-rate drying period when *a*_*w*_ < 1.

Cycle	Average water mass loss during linear drying (g)
1	0.73 ± 0.04^a^
2	0.73 ± 0.04
3	0.68 ± 0.07
4	0.74 ± 0.02
5	0.76 ± 0.01

a 95% confidence interval.

### 3.3 Clogging results

**Fig. 6:**
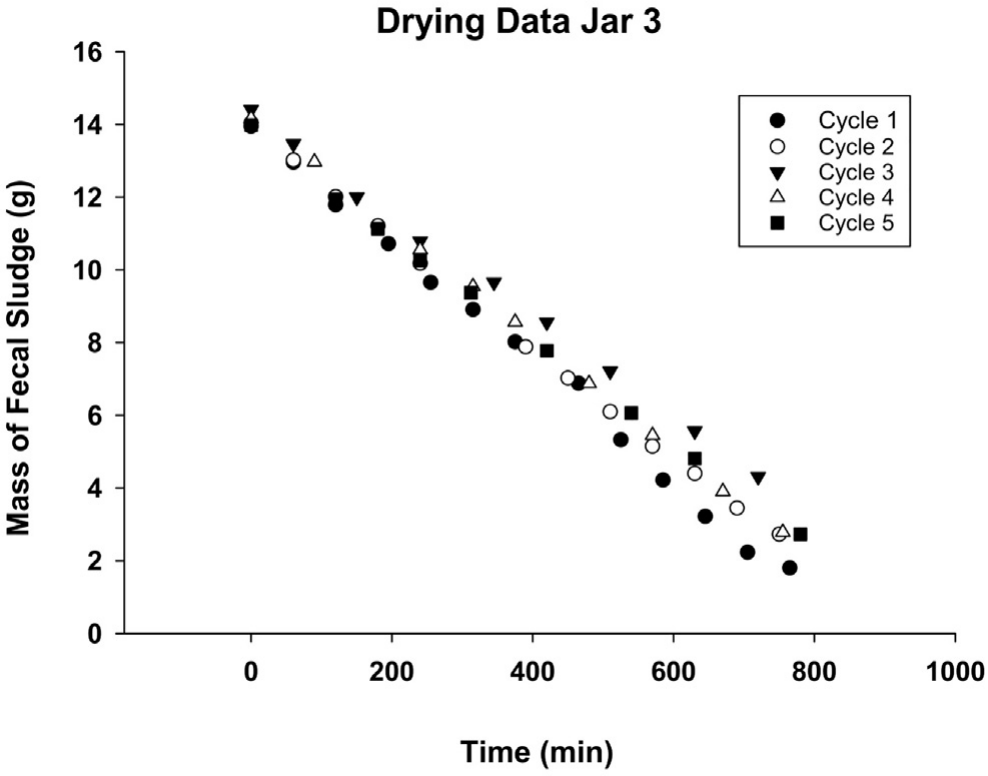
Drying date for FS contained in Jar 3 for five cycles. Variations in drying rates between cycles are associated with variability in *RH* and temperature inside the growth chamber and temperature variability inside the jar.

To assess potential clogging and loss of drying performance through usage, FS drying curves from the five experimental cycles were compared. Only data above the critical moisture content were used, as these are in the constant-rate drying period when the assumption of saturated water vapor pressure on the FS side of the laminate is reasonable. Fig. 6 depicts these data for Jar 3 for the five drying cycles. The data indicate that the drying rate was similar for Cycles 1, 2, 4, and 5, but somewhat faster for Cycle 3. While the slower drying rate for Cycles 2, 4, and 5 might be due to pore clogging from dried biosolids deposited during previous cycles but not washed away during rinsing, it may also be due to small differences in *RH* and temperature between the cycles. Environmental conditions for Jar 3 during Cycle 3 include the temperature inside the jar and *RH* and temperature outside the jar and are plotted in Fig. S1, S2, and S3, respectively, in Supplementary Material. Over the course of Cycle 3 these parameters varied by 3.3, 6.25 and 3.5% for temperature inside the jar, *RH*, and temperature outside the jar, respectively.

**Fig. 7:**
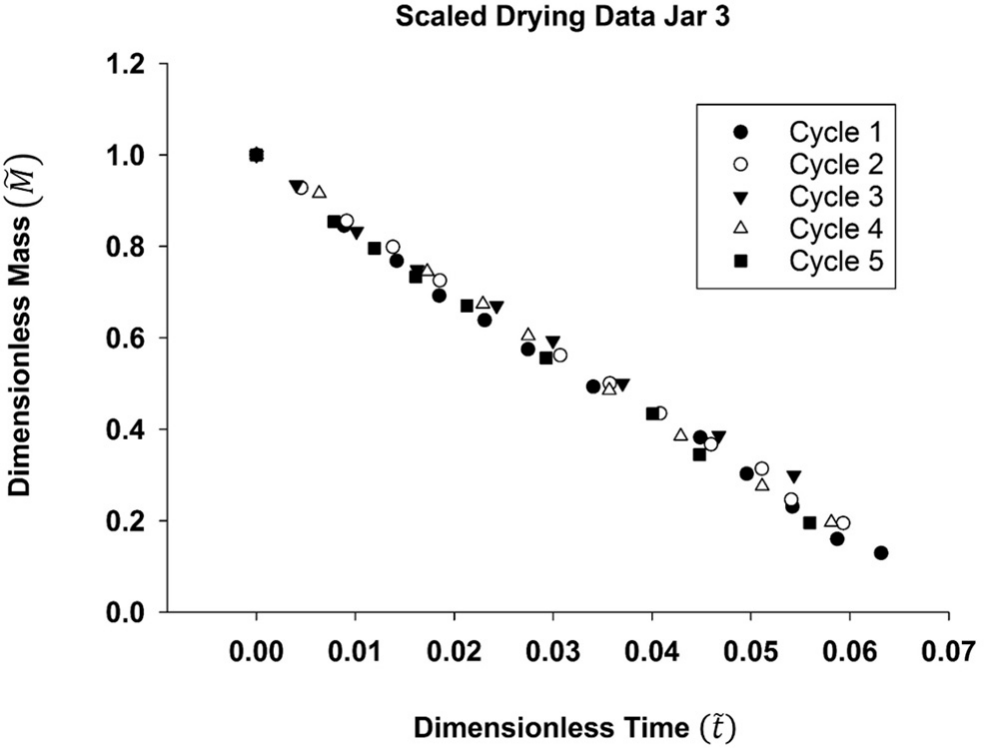
Scaled data for FS drying over five cycles in Jar 3. After scaling the raw data, the effects of *RH* and temperature variability within the growth chamber and jar were removed.

To correct for variations in environmental conditions, data were scaled according to the approach described in Section 1.2, using measured *RH* and temperatures for each experiment and assuming *RH* = 100% at the FS/laminate interface on the inside of the jar. Scaled drying data for Jar 3 are shown in Fig. 7: most of the variability observed in the raw data for the five cycles in Fig. 5 is removed with scaling.

Using these scaled data, analysis of covariance (ANCOVA) (Huitema, 1980) was used to test the hypothesis that the drying rate, the slope *ε*/*τ* in Eq. (4), was independent of experiment cycle. For each jar, ANCOVA was used to compare the constant drying rates for the five cycles, the slopes of regression lines to scaled data, by testing the effect of a categorical factor, experimental cycle, on dependent variable, dimensionless mass, while controlling for the effect of a continuous co-variable, dimensionless time. The categorical factor splits the relationship between continuous co-variable and dependent variable into several linear regressions, one for each level of the categorical factor (Huitema, 1980; Montgomery et al., 1984). At a significance level of *α* = 0.05, the hypothesis of a meaningful change in performance of the eVent laminate because of clogging over five cycles was rejected, since the slopes of the regression lines (drying rates, *ε*/*τ*) were not different with *P*-value = 0.35, 0.45 and 0.44 for Jars 1, 2 and 3 respectively. Thus, there was no significant reduction in the performance of the laminate due to repeated usage with minor brushing/rinsing between uses. Fitted *ε*/*τ* for the linear segment of drying curves are presented in Table 2.

**Table 2:** Slope of scaled drying data, *ε*/*τ*, for three FS-filled jars for all drying cycles.

Jar	Fitted *ε*/*τ*				
	Cycle 1	Cycle 2	Cycle 3	Cycle 4	Cycle 5
1	0.117 ± 0.009	0.112 ± 0.005	0.107 ± 0.004	0.102 ± 0.003	0.114 ± 0.005
2	0.108 ± 0.011	0.113 ± 0.003	0.096 ±0.002	0.108 ± 0.003	0.108 ± 0.005
3	0.112 ± 0.007	0.111 ± 0.003	0.107 ± 0.005	0.120 ± 0.001	0.112 ± 0.009

While clogging of the eVent laminate was not observed for FS drying over five cycles in this study, membrane clogging is one of the primary obstacles impeding the application of membrane distillation for the recovery of ammonia from swine manure (Zarebska et al., 2014). In that application, gradients in ammonia vapor pressure are used to extract ammonia from swine manure in systems that are operated continuously (Xie et al., 2016). Typically, only minor reductions in ammonia flux are observed over the first few days. Fouling occurs slowly as nonvolatile constituents in the swine manure slowly adsorb onto the hydrophobic membrane. Recent studies indicate that organic peptides and proteins that adsorb onto hydrophobic surfaces were the primary cause of fouling, slowly causing the membrane surface to change from hydrophobic to hydrophilic (Thygesen et al., 2014; Xie et al., 2016). It is possible that a similar clogging process may occur for FS drying over longer time periods than examined in this study, which may permit the slow adsorption of contaminants onto the hydrophobic membrane that gradually changes the surface wettability. Future work should explore this.

The effective diffusion length for FS and DI drying experiments in the eVent laminate (see Eq. (1)) can be determined from the constant drying rate data from all jars and over all five cycles, since there was no significant reduction in performance with cycle. Best fit *λ* were determined using Solver in Microsoft Excel using the macro SolverAid (Marzooghi et al., 2017): *λ*_*FS*_=4.43 ± 0.21 × 10^−3^ (m) and *λ*_*DI*_=3.94 ± 0.11 × 10^−3^ (m). The larger *λ* for FS than DI water may be due to the deposition and penetration of fecal matter on the surface of the laminate during drying, which increases the effective diffusion length and slows drying.

Marzooghi et al. (2017) determined that the best-fit effective diffusion length for GORE Wrap Cover Laminate was *λ*_*DI*_=9.92 ± 0.10 × 10^−3^ (m), which is 2.5 times larger than that for the eVent laminate in this study: *λ*_*DI*_=3.94 ± 0.11 × 10^−3^ (m). Larger *λ*_*DI*_ for GORE versus eVent laminate indicates slower drying and is likely due to the hydrophobic fabric in contact with DI water for the GORE laminate, while the hydrophilic fabric contacted DI water for experiments with the eVent laminate. Pores in the hydrophobic fabric of the Gore laminate remained air-filled while pores in the hydrophilic fabric of the eVent laminate were water filled. Thus, the diffusive pathway for water vapor transport was likely longer for the Gore than the eVent laminate. In addition, the tortuosity may have been larger or porosity smaller for the GORE versus the eVent laminate.

For biosolids drying in the GORE Wrap Cover Laminate, *λ*_*AD biosolid*_ GORE/*λ*_*DI*_ GORE=1.26 for anaerobically digested biosolids (Marzooghi et al., 2017). Similarly, *λ*_*FS*_ eVent/*λ*_*DI*_ eVent=1.12 for FS drying in the eVent laminate. These results suggest that while the effective diffusion length is different for DI water versus sludge drying in laminated hydrophobic membranes, experiments of DI water evaporation through laminates may be used to estimate corresponding sludge drying (anaerobically digested biosolids or FS) by increasing *λ*_*DI*_ by 10–30% to account for the effect of biosolids on reducing drying rate. Experiments in other laminated hydrophobic membranes are needed to confirm this hypothesis.

### 3.4 Scanning electron microscopy and energy dispersive X-ray analyses

**Fig. 8:**
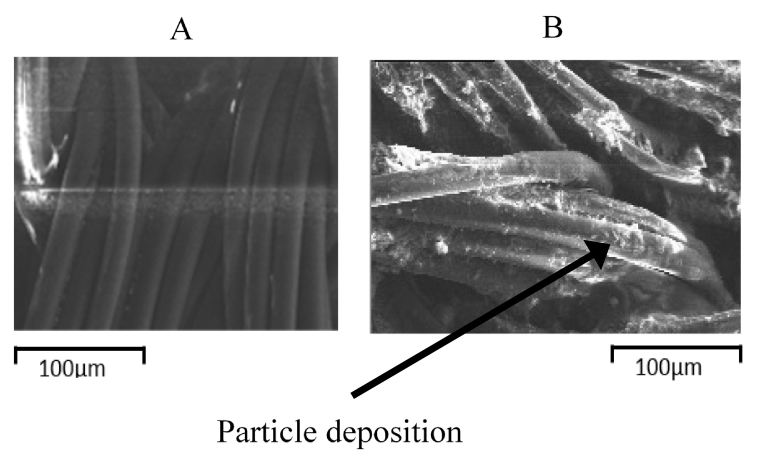
SEM images of the eVent laminate's inner fabric: new (A), and used/rinsed (B) after contact with FS for five drying cycles.

While significant reduction in FS drying through the laminate was not observed over five cycles of drying, FS contains dissolved and colloidal organic and inorganic compounds that may accumulate on the laminate fabric and through time alter laminate performance. SEM images of the inner hydrophilic fabric, which is in contact with FS during experiments, of new and used/rinsed eVent laminate after five cycles of use are shown in Fig. 8. The surface of the new laminate is smooth, while the used/rinsed surface is rough. The rougher surface of used/rinsed laminate is due to particles accumulating on the FS-side of the laminate.

**Fig. 9:**
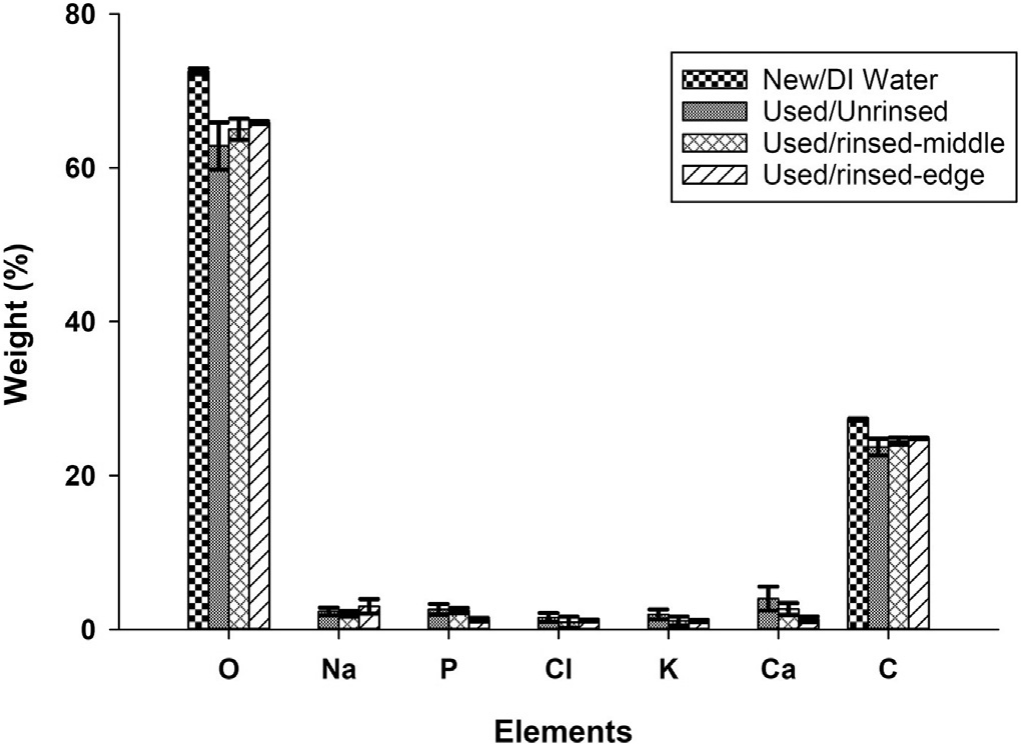
Elemental analysis of the inner fabric of eVent laminate for samples collected from the middle and edge of new, used, and used/rinsed eVent laminate samples. (Error bars indicate 95% confidence intervals).

EDX results of the inner hydrophilic fabric of new, used and used/rinsed laminates are shown in Fig. 9. The most significant element detected for all eVent laminates (new, used and used/rinsed) was O. The other significant element detected is C, whose weight normalized to the total weight of elements is 23.7% and 24.5% for middle of the used and the middle of the used/rinsed laminate, respectively, and 27.3% on the new laminate where only evaporation of DI water through the laminate occurred. Trace amounts of Na, P, Cl, K and Ca were detected on the inner fabric of the used and used/rinsed laminates, while none of these elements were detected on the inner fabric of the new laminate. O and C were the only elements measured on the inner fabric of the new laminate.

Even though mild brushing/rinsing was unable to prevent accumulation of trace amounts of elements on the inner fabric of the laminate, the eVent laminate's performance did not deteriorate through five cycles of FS drying. This could be because deposition of the elements on the inner fabric of the laminate was not sufficient to clog the large inner fabric pores (~25 μm estimated from SEM images), and deposition onto the surface of the membrane with much smaller pores (0.14 μm) was not significant enough to cause clogging. Thus, the inner fabric appeared to protect the membrane from clogging, at least for five cycles of FS drying. As discussed earlier, while laminate clogging was not significant enough to alter FS drying rates here, longer-term experiments should be conducted in the future.

### 3.5 Water pressure effect on drying rate

**Fig. 10:**
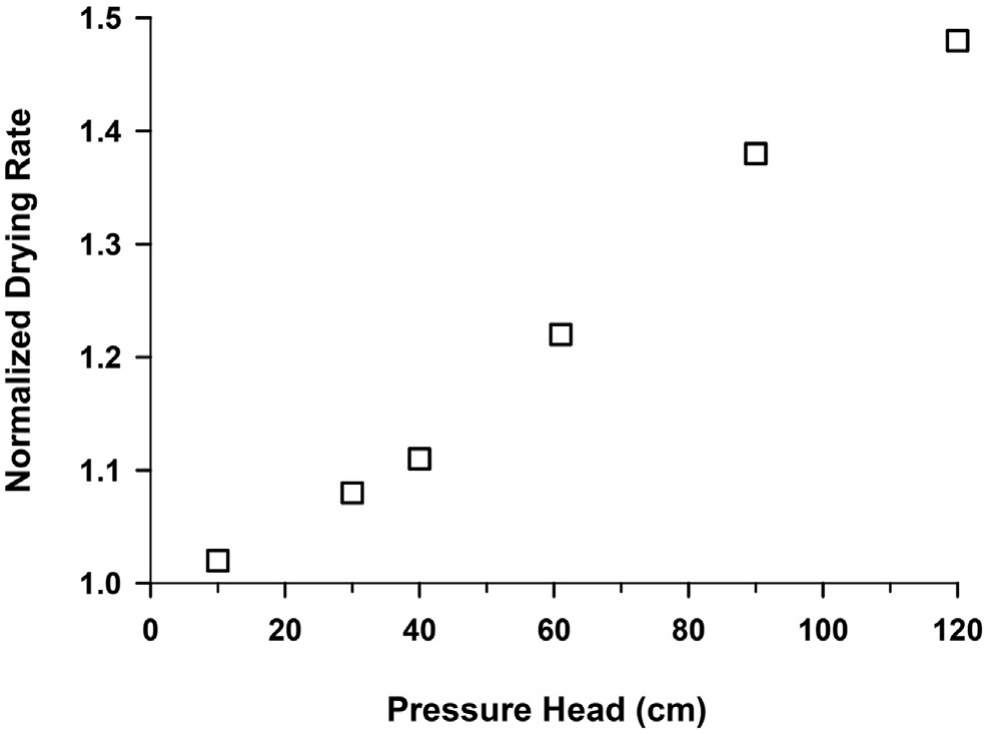
Normalized drying rate of water through the eVent laminate as a function of water pressure head.

The effect of water pressure head on drying rate is shown in Fig. 10, where the drying rate at 10, 30, 40, 60, 90 and 120 cm water pressure heads are normalized with respect to 1 cm pressure head. As water pressure exerted on the eVent laminate increases, the drying rate increases linearly. The drying rate at 10 cm water pressure head is approximately 7% higher than that at 1 cm pressure head, while the drying rate at 120 cm is about 50% greater than that at 1 cm. We postulate that the water pressure effect is due to compression of the thin laminate, which reduces the water vapor diffusion length (see Fig. 1). For toilets lined with laminated hydrophobic membranes on the bottom and sides, the water flux through the bottom of the toilets may be significantly larger than through upper laminate surfaces.

## 4 Conclusions

A decentralized container-based sanitation system may be enhanced with laminated hydrophobic membranes, which contain and enhance drying of FS in toilets. The laminated hydrophobic membrane is hydrophobic and only allows vapor transport, which is driven by moderate temperature or humidity gradients, while other constituents, including both particulate and dissolved matter are retained. A critical issue concerning this application is laminate clogging after repeated use. Clogging might reduce performance and decrease replacement interval, thus limiting the benefits of laminate application.

Controlled laboratory experiments were conducted to evaluate laminate clogging after repeated use during FS drying. Over five drying cycles with mild brushing/rinsing of laminates between cycles, at a significance level of *α* = 0.05 the dimensionless drying rate was not reduced. While scanning electron microscopy and energy dispersive X-ray analyses of a used laminate showed deposition of fecal matter on the inner fabric of the laminate in contact with FS, particulate accumulation was never sufficient to alter FS drying rate. These data demonstrate that clogging of the laminated hydrophobic membrane is minor over five cycles of use with mild rinsing/brushing between cycles. Further, the results suggest that laminated hydrophobic membranes might be used repeatedly to contain FS without unwanted clogging in field applications.

The laboratory experiments also showed that FS dried at a constant, linear rate during all experiments, but slowed when the moisture content dropped below a critical value (11.6 (g/g)), when water activity <1. The ratio of the effective diffusion length through the laminated hydrophobic membrane for FS or anaerobically digested biosolids drying to DI water evaporation was ≈1.1 − 1.3 during the constant-rate drying period. Thus, experiments of DI water evaporation might be used to estimate biosolids drying in the same laminates by increasing the effective diffusion length by 10–30%. Although this result was shown to be valid for only two laminates, the data suggest scaling water evaporation results to biosolids drying might allow rapid estimate of FS drying in laminates without the difficulty of FS drying experiments.

The experimental results also demonstrate that water pressure can increases the drying rate in laminate-lined toilets in CBS systems: water mass flux is increased by up to 50% for water pressures heads of 120 cm. Thus, FS drying might be enhanced at the bottom of laminate-lined toilets in CBS systems, suggesting that it may be important to ventilate the bottom of such toilets to maximize FS drying.

## Appendix A Supplementary Data

Supplementary material Image 1
