# Supplementary material for: Assessing clogging of laminated hydrophobic membrane during fecal sludge drying
Source: Sci Total Environ. 2018 Jun 15;627:713–22. doi: 10.1016/j.scitotenv.2018.01.209 (PMC5892458; doi:10.1016/j.scitotenv.2018.01.209)
Supplement: Supplementary file 1 — Supplementary material [file mmc1.docx]

**Supplementary Material**


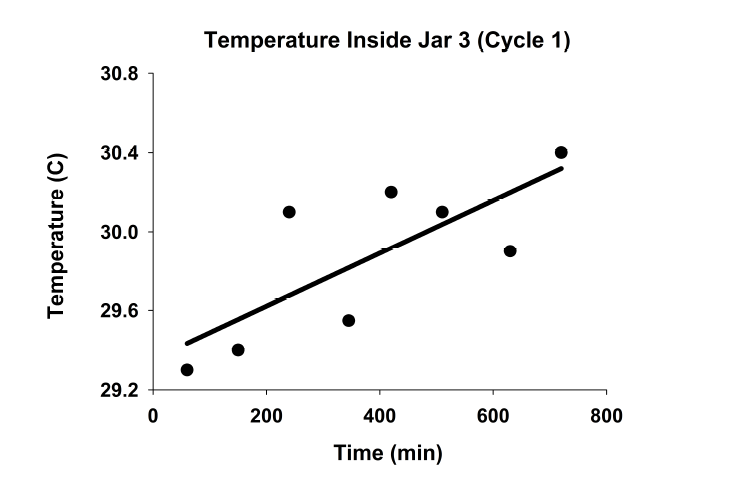


Fig. S1. Temperature variation inside Jar 3 (Cycle 1) during the FS drying experiment. To scale the raw data, the regression line was used to estimate the temperature inside Jar 3 during the experiment.


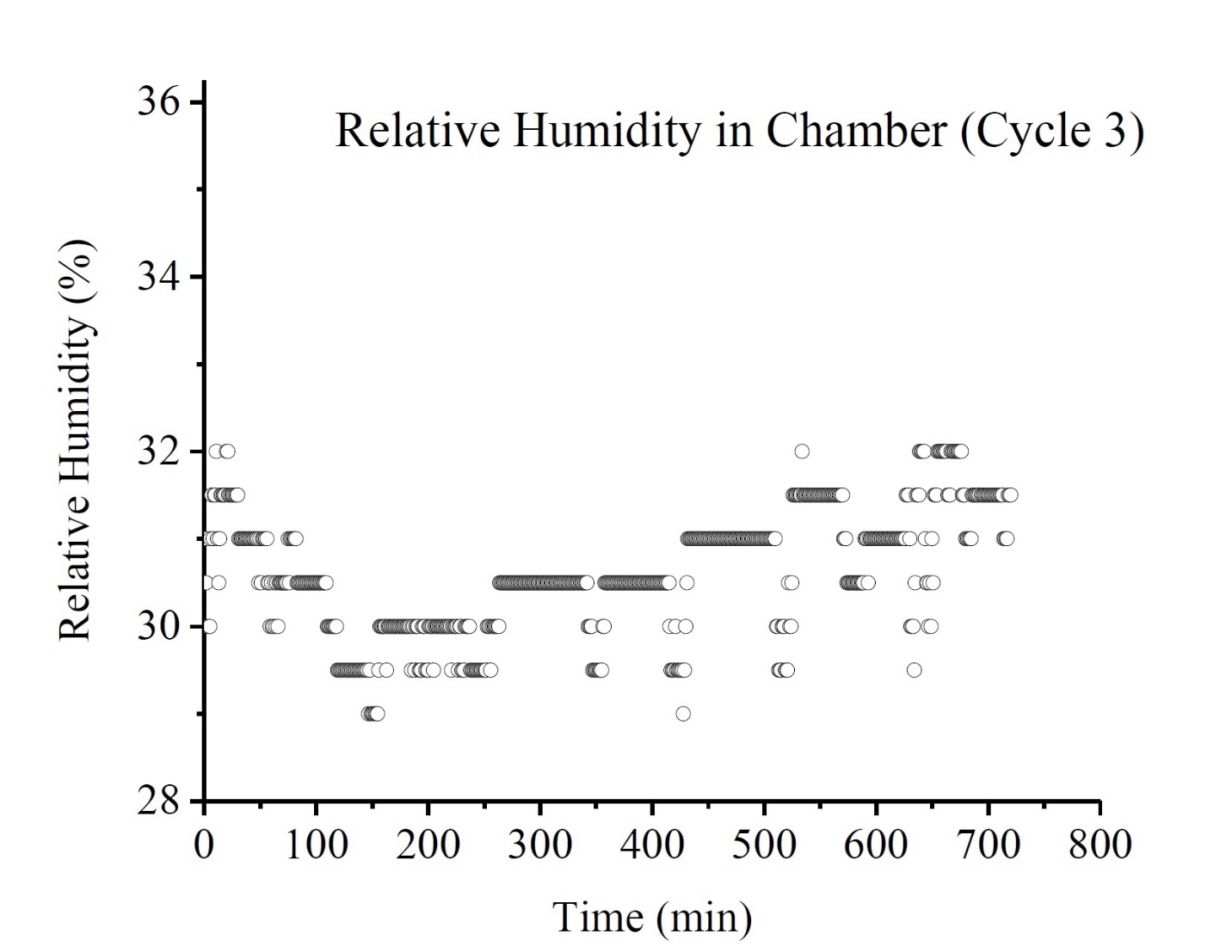


Fig. S2. Relative humidity variation inside the growth chamber for Cycle 3 experiments. To scale the raw data, the relative humidity at each measurement time was used.


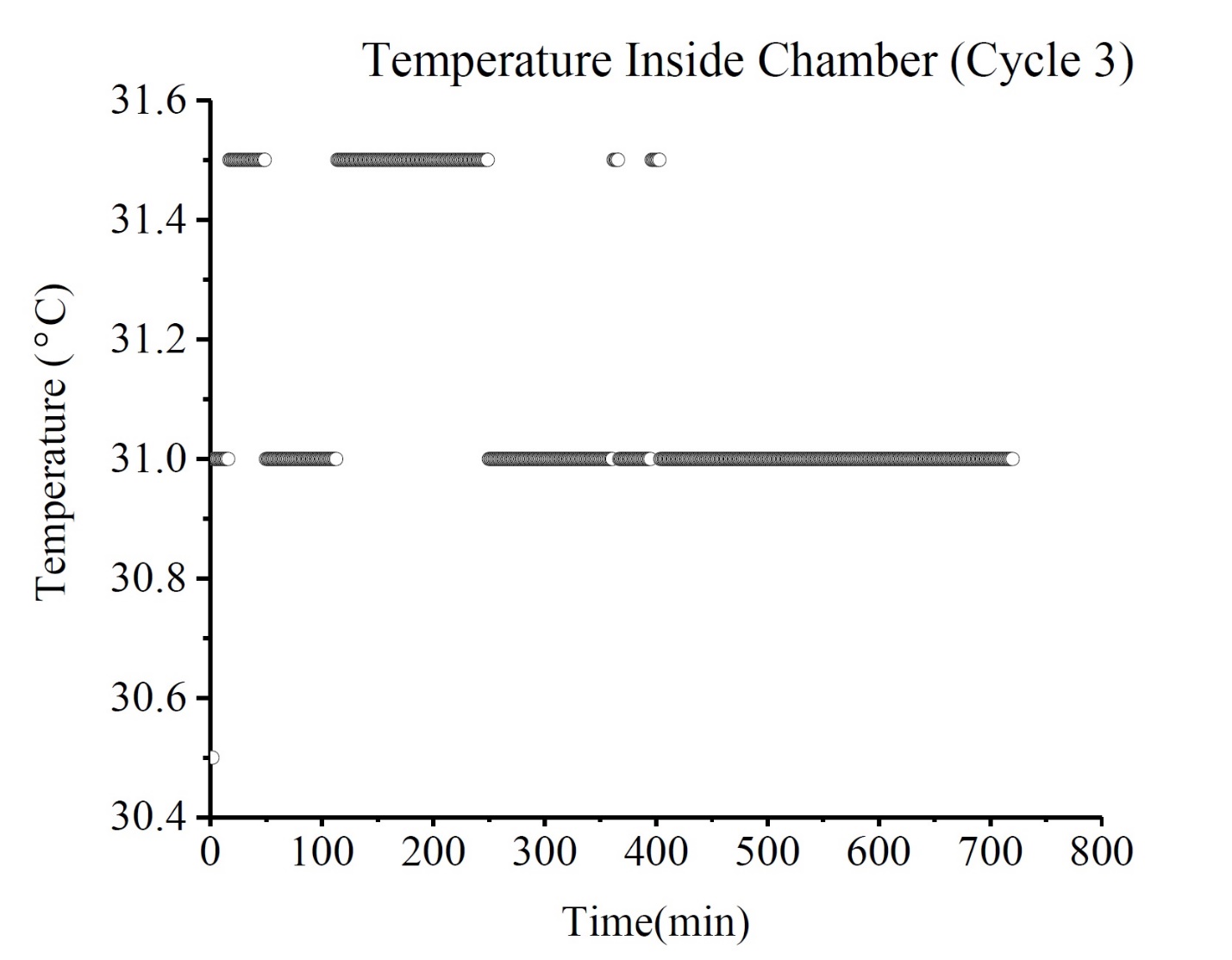


Fig. S3. Temperature variation inside the growth chamber for Cycle 3 experiments. Temperature at each measurement time was used to scale the raw data.
